# Decreased Cytoplasmic Expression of ADAMTS14 Is Correlated with Reduced Survival Rates in Oral Squamous Cell Carcinoma Patients

**DOI:** 10.3390/diagnostics10020122

**Published:** 2020-02-23

**Authors:** Yueh-Min Lin, Chiao-Wen Lin, Jeng-Wei Lu, Kun-Tu Yeh, Shu-Hui Lin, Shun-Fa Yang

**Affiliations:** 1 Department of Pathology, Changhua Christian Hospital, Changhua 500, Taiwan; 93668@cch.org.tw (Y.-M.L.); 10159@cch.org.tw (K.-T.Y.); 2 Department of Medical Laboratory Science and Biotechnology, Central Taiwan University of Science and Technology, Taichung 406, Taiwan; 3 School of Medicine, Chung Shan Medical University, Taichung 402, Taiwan; 4 Institute of Oral Sciences, Chung Shan Medical University, Taichung 402, Taiwan; cwlin@csmu.edu.tw; 5 Department of Dentistry, Chung Shan Medical University Hospital, Taichung 402, Taiwan; 6 Department of Biological Sciences, National University of Singapore, Singapore 117543, Singapore; jengweilu@gmail.com; 7 Institute of Medicine, Chung Shan Medical University, Taichung 402, Taiwan; 8 Department of Medical Research, Chung Shan Medical University Hospital, Taichung 402, Taiwan

**Keywords:** ADAMTS14, immunohistochemistry, tissue microarray, oral squamous cell carcinoma, survival

## Abstract

A disintegrin and metalloproteinase with thrombospondin motif 14 (ADAMTS14) is a member of the zinc-dependent protease family that is implicated in the occurrence and progression of tumors. Oral cancer (OC) is a common cancer worldwide, but it is particularly prevalent in Taiwan. However, whether the expression of ADAMTS14 is correlated with the carcinogenesis and progression of oral squamous cell carcinoma (OSCC) has not yet been investigated. In this study, we used immunohistochemistry (IHC) to examine 250 OSCC specimens in order to identify correlations between the cytoplasmic expression of ADAMTS14 and (1) clinicopathological features of OSCC as well as (2) clinical outcomes of OSCC. Our results indicate that cytoplasmic expression of ADAMTS14 was lower in OSCC tissues than in normal tissues. In analyzing correlations between ADAMTS14 expression and clinicopathological features, we found that negative cytoplasmic expression of ADAMTS14 was significantly associated with higher frequencies of lymph node metastasis and more advanced AJCC stages (III/IV). Kaplan–Meier survival analysis revealed that negative cytoplasmic expression of ADAMTS14 was also associated with significantly worse OSCC survival. Univariate and multivariate analyses confirmed that cytoplasmic expression of ADAMTS14 was associated with lymph node metastasis, tumor stage, and tumor grade and also indicated that cytoplasmic ADAMTS14 expression may be an independent prognostic factor for OSCC. This is the first study to report that the cytoplasmic expression level of ADAMTS14 is associated with OSCC prognosis and tumor progression. Our data indicate that ADAMTS14 can serve as a prognostic marker and a potential therapeutic target for OSCC.

## 1. Introduction

Oral cancer (OC) is a type of head and neck cancer, and the main sites of OC are often located in the lips, tongue, mouth, cheeks, gums, or palate [1]. Globally, OC is the sixth most common cancer, causing more than 145,000 deaths per year; however, OC is more common in men who smoke and drink excessively [2]. Oral squamous cell carcinoma (OSCC) is a type of malignant cancer that is common in Taiwan [3], India, and other Southeast Asian countries [4]. The primary external risk factors for OSCC are smoking, drinking, consumption of smokeless tobacco, consumption of betel nuts, and infection with human papillomavirus (HPV) [5,6]. Intrinsic factors which cause normal cells to become tumor cells include (1) genetic and epigenetic changes [7] and (2) the post-translational modification (PTM) (i.e., phosphorylation, glycosylation, and ubiquitination) of specific proteins [8]. Recently, more than 300,000 cases of OC have been newly diagnosed, OC-related morbidity and mortality have increased in younger populations [2], and more than 50% of OSCC patients have been found to have a high rate of lymph node metastasis (LNM) [9]. Despite recent advances in radiation therapy, chemotherapy, and traditional surgery, once an OSCC patient is diagnosed with LNM, the 5-year survival rate drops from 80% to less than 40% [10,11]. One reason that OSCC prognosis is poor is due to a lack of reliable, early biomarkers, which limits the use of highly accurate drugs. Therefore, identifying reliable biomarkers that can be detected early could greatly benefit OSCC treatment.

In humans, a disintegrin and metalloproteinase with thrombospondin motifs (ADAMTS) are enzymes which belong to the extracellular zincmetallo proteinase family. This family is comprised of 19 members, including ADAMTS1, 4, 5, 8, 9, 15, and 20 (aggrecanases or proteoglycanases); ADAMTS2, 3, and 14 (procollagen N-propeptidases); ADAMTS7 and 12 (cartilage oligomeric matrix protein-cleaving enzymes); ADAMTS13 (Von-Willebrand Factor proteinase); and ADAMTS6, 10, 16, 17, 18, and 19 (a group of orphan enzymes). These enzymes, which are secreted during tissue morphogenesis and during the pathophysiological remodeling of multi-domain matrix-associated zinc, have multiple roles. Evidence indicates that the ancillary domains of ADAMTS enzymes have important regulatory roles in propeptide processing and glycosylation and that ADAMTS proteins have regulatory roles which control the structure and function of the extracellular matrix (ECM) [12]. Enzymes which catalyze multiple substrates in the ECM via ADAMTS domains are known as multidomain proteases [13]. ADAMTS proteases play an important role in tissue development and maintenance, and disorders and mutations in these proteases are associated with various diseases [12]. Recent evidence suggests that ADAMTS proteases are involved in arthritis [14,15], cancer [16,17,18,19,20], atherosclerosis [21], fertility issues, defective wound healing, defective angiogenesis, and central nervous system damage/disorders [22,23].

ADAMTS14 is a member of the ADAMTS zinc-dependent protease family located on chromosome 10q22.1 [12,13,24,25], which contains an N-terminal catalytic domain and a C-terminal ancillary domain, which defines substrate specificity, including thrombospondin type 1 repeats (TSR) domain [13] and a unique proline-rich region (PRR) have an important role in identifying its procollagen substrate [26,27]. ADAMTS14 has sequence and functional homology with ADAMTS2 and ADAMTS3. These enzymes share similar activities and are characterized by the presence of four thrombospondin type I (TSR1) domains and a C-terminal procollagen N-proteinase (PNP) domain, which contain a protease and lacunin (PLAC) domain. ADAMTS2 has been identified as a potential receptor which mediates antiangiogenic properties by interrupting intratumoral vascularization [28]. Conversely, although low levels of ADAMTS14 are usually co-expressed with ADAMTS2 in all type I, collagen-rich connective tissues, it also functions as a trigger for ECM remodeling [29]. 

ADAMTS14 has been shown to be associated with several cases of genetics, and ADAMTS1 gene polymorphisms have been found to influence genetic susceptibility to multiple sclerosis [30]. An ADAMTS14 gene variant has also been shown to be a risk factor for knee osteoarthritis and Achilles tendon pathologies [31,32,33]. Recently, there has been a surge in reports on how ADAMTS proteases affect the tumor microenvironment to enhance cancer progression [34]. For example, the expression of ADAMTS14 is now known to be significantly upregulated in human breast carcinomas tissues [35]. Furthermore, gene polymorphisms of ADAMTS14 were recently found to regulate (1) the impact of smoking on the clinicopathological development of hepatocellular carcinoma (HCC) [36] and (2) the influence of environmental risk factors on the development of OC [34]. 

Nonetheless, it is important to better elucidate the role of ADAMTS14 expression and other clinical factors in OSCC. In the current study, we examined expression of ADAMTS14 proteins in 250 OSCC tissues using immunohistochemistry (IHC). We also analyzed associations between ADAMTS14 protein expression with clinicopathological features of OSCC and with OSCC prognosis. Finally, we sought to identify promising prognostic indicators in order to facilitate early detection and timely treatment for OSCC patients in the future.

## 2. Results

### 2.1. Clinical Prameters of OSCC Patients

To determine the role of ADAMTS14 in OSCC, we first examined the expression level of ADAMTS14 in OSCC tissues using IHC staining. Table 1 summarizes the clinicopathologic data from the 250 OSCC patients whose tissues were included in this study. Normal oral mucosa tissues from OSCC patients were used as a control. Among the OSCC patients, 238 (95.2%) were male and 12 (4.8%) were female. The average age of patients was 54.9 years (range: 31–88 years). With regard to AJCC cancer stage, 46 patients (18.4%) were in stage I, 54 (21.6%) were in stage II, 28 (11.2%) were in stage III, and 122 (49.0%) were in stage IV. Histological grade analysis identified 42 (16.8%) well-differentiated (WD) cases, 201 (80.4%) moderately differentiated (moderate) cases, and 7 (2.8%) poorly differentiated (poor) cases. With regard to clinical treatment, 152 patients (60.8%) were undergoing radiotherapy (RT), and 60 (24.0%) were undergoing chemotherapy (CT). 

**Figure 1:**
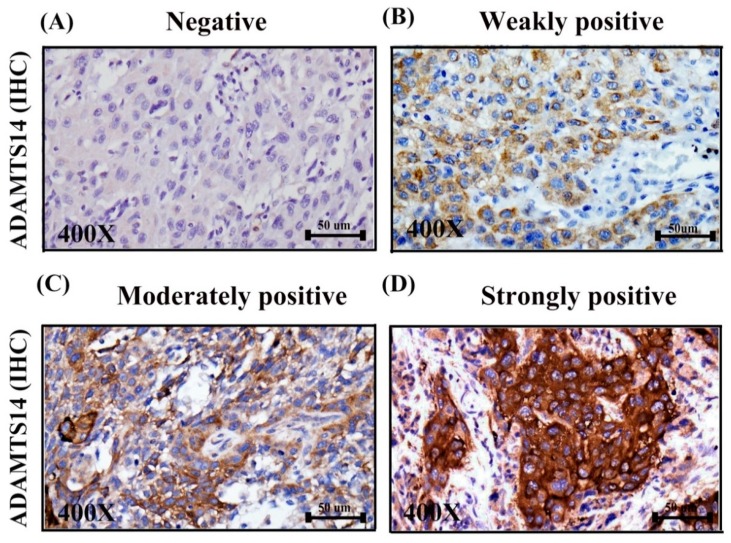
Expression of a disintegrin and metalloproteinase with thrombospondin motif 14 (ADAMTS14) in OSCC as detected by immunohistochemical staining. (**A**). Negative expression of ADAMTS14 in OSCC tissues. (**B**). Weakly positive cytoplasmic expression of ADAMTS14 in OSCC tissues. (**C**). Moderately positive cytoplasmic expression of ADAMTS14 in OSCC tissues. (**D**). Strongly positive cytoplasmic expression of ADAMTS14 in OSCC tissues (magnification 400×).

ADAMTS14 proteins were found to be expressed in the cytoplasm of tumor cells. The positive expression of protein was further divided into three types: score 1+ indicated weak positive expression; score 2+ indicated moderately positive expression, and score 3+ indicated strongly positive expression. Negative ADAMTS14 expression was detected in tissues from 52 of the 250 patients (20.8%), and positive ADAMTS14 expression was detected in tissue from 198 of the 250 patients (79.2%), as shown in Figure 1. Patient characteristics, including age, histological grade, T status, lymph node metastasis, distant metastasis, AJCC cancer stage, frequency of drinking, frequency of betel nut chewing, RT, CT, and survival, are listed in Table 1.

### 2.2. Correlations between ADAMTS14 Expression and ClinicopathologicF of OSCC

Correlations between ADAMTS14 expression and clinicopathologic features of OSCC were analyzed by IHC. We categorized tissue samples as having either negative (*n* = 52) or positive (*n* = 198) ADAMTS14 expression based on IHC staining intensity. We further divided the positive and negative expression groups into subgroups, whereby the positive expression group included tissues with a score of 1+ or 2+ and the negative expression group included tissues with − or +/− scores. Correlations between ADAMTS14 expression and clinicopathologic features are summarized in Table 2. Negative cytoplasmic expression of ADAMTS14 was significantly correlated with lymph node metastasis (*p* = 0.003) and AJCC cancer stage (*p* = 0.031) using Fisher’s Exact Test or Chi-square test. However, no significant differences were found between ADAMTS14 expression and age, histological grade, T status, distant metastasis, frequency of drinking, frequency of betel nut chewing, or survival.

### 2.3. Negative Cytoplasmic Expression of ADAMTS14 was Associated with Short Overall Survival in OSCC Patients

**Figure 2:**
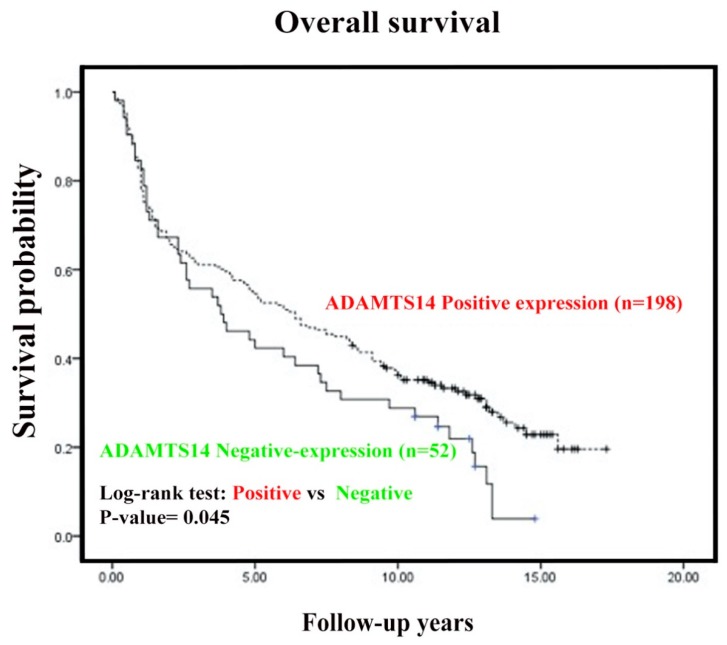
Influence of ADAMST14 expression on Kaplan–Meier survival curves among OSCC patients. Kaplan–Meier overall survival curves revealed survival differences between OSCC patients with positive cytoplasmic expression of ADAMTS14 and negative cytoplasmic expression of ADAMTS14 was also calculated using the log-rank tests for the homogeneity of Kaplan–Meier curves.

We further evaluated the correlation between ADAMTS14 expression and patient overall survival. Kaplan Meier analysis showed that OSCC patients with negative ADAMTS14 expression had worse survival curves than did patients with positive ADAMTS14 expression (*p* = 0.045) using log-rank tests (Figure 2).

### 2.4. Prognostic Variables in OSCC Patients Determined According to Cox Proportional Hazard Model Analysis

Univariate analysis demonstrated that patients with negative ADAMTS14 expression had a 1.4-fold higher hazard risk than patients with positive AMADTS14 expression (95% CI, 0.507–0.996; *p* = 0.047). As compared to patients with AJCC stage and negative ADAMTS14 expression, the mortality hazard risk was multiplicatively enhanced among patients with lymph node metastasis (95% CI, 1.451–2.556; *p* < 0.001, HR = 1.9), advanced cancer stage (95% CI, 1.312–2.361; *p* < 0.001, HR = 1.8), and moderate/poor tumor differentiation (95%, 1.216–2.860, *p* = 0.004, HR = 1.9). Subsequent multivariate analysis revealed that lymph node metastasis (95% CI, 1.004-20.42; *p* = 0.048, HR = 0.048), AJCC advanced cancer stage (95% CI, 1.065–2.183; *p* = 0.021, HR = 1.5), moderate/poor tumor differentiation (95% CI, 1.191–2.943; *p* = 0.007, HR = 1.9) and ADAMTS14 negative expression (95% CI, 0.601–1.199; *p* = 0.0353, HR = 1.2). All these findings suggest that AMADTS14 is an independent prognostic factor in oral cancer patients.

## 3. Discussion

OSCC is one of the top ten malignant tumors in the world and is a widely recognized health problem. Improvements in surgical techniques have not improved the morbidity and mortality rates associated with OSCC [9]. Indeed, despite many recent advances in a variety of treatment modalities, the overall survival rate of OSCC has remained unchanged at 50% over the past four decades. Complex invasiveness and a high recurrent rate are the main causes of treatment failure and poor prognosis for OSCC [37]. To date, treatment strategies and prognostic determinations have largely depended on diagnostic staging and histopathological criteria. However, accurate prognoses cannot be determined based on existing tumor staging and classification systems. These factors underscore the importance of identifying biomarkers which can assess the risk of poor prognosis during the early stages of cancer [38,39]. At present, there are few biomarkers or targets for OSCC diagnosis and treatment. Therefore, identifying genes which have different expression patterns in OSCC tumors and normal tissues should help elucidate the pathogenesis of OSCC and may provide useful diagnostic biomarkers and therapeutic targets for OSCC treatment [40].

A growing body of evidence has suggested that ADAMTS proteins play a role in malignancies, tumor progression, cell proliferation, apoptosis, migration, invasion, and angiogenesis [41,42,43,44]. Although the exact mechanism by which ADAMTS affects tumor progression and metastasis is unclear, many studies have investigated these proteolytic enzymes and determined the roles they play in different types of tumors. However, results have been varied and sometimes contradictory. The function of these functions depends or independent of their angiogenic activity. For example, some members of this proteinase family have different expression patterns in normal tissues and tumors tissues. ADAMTS1, ADAMTS3, ADAMTS5, ADAMTS8, ADAMTS9, ADAMTS10, ADAMTS15, and ADAMTS18 have been found to be significantly downregulated in tumors [17], whereas ADAMTS4, ADAMTS6, and ADAMTS14 have been found to be significantly upregulated in tumors [45]. Conversely, some members of the ADAMTS family have been reported to have anti-angiogenic effects and found to be epigenetically inhibited in various types of cancer [46]. ADAMTS1 affects the tumor microenvironment in a manner unrelated to angiogenesis. Specifically, ADAMTS1 increases tumor growth rate by inhibiting Matrigel and fibrin gel function [45]. ADAMTS belongs to the adamalysin family and is also found in snake venom proteases, many of which are involved in loops of interaction with certain inflammatory mediators, including tumor necrosis factor-alpha (TNF-α) [47], transforming growth factor-alpha (TGF-α), and transforming growth factor-beta (TGF-β) [48,49]. In vivo, experiments involving Adamts2-Adamts14-deficient mice demonstrated that ADAMTS plays an important role in immune system regulation, possibly through crosstalk between mesenchymal cells and the TGF-β pathway [50].

Other studies have reported that ADAMTS3 and ADAMTS14 are homologs of ADAMTS2 and that these enzymes share similar biochemical functions. ADAMTS3 and ADAMTS14 are involved in the processing of type II procollagen N-propeptidase [51] and type I procollagen N-propeptidase [24], respectively. In gastric cancer, overexpression of ADAMTS2 is associated with poor clinical prognosis [52]. ADAMTS2 is also a potential marker of dysplasia of craniofacial fibrous [53]. SP1-related transcriptional regulation of ADAMTS3 gene expression in osteosarcoma cell models [54]. Furthermore, the competing endogenous circular ADAMTS14 might suppress HCC progression through regulating miR-572/RCAN1 as the competing endogenous RNA [55]. Nonetheless, to the best of our knowledge, this is the first study to investigate the role of ADAMTS14 protein expression in OSCC and also the first study to investigate the association between ADAMTS14 protein expression and other clinicopathological features of OSCC. Our IHC results revealed that ADAMTS14 proteins are expressed in OSCC tissues. Specifically, we found that that cytoplasmic expression of ADAMTS14 proteins was significantly lower in OSCC tissues than in normal oral mucosa control tissues (Figure 1).

After analyzing 250 OSCC tissue samples using IHC, we used clinical information to investigate correlations between ADAMTS14 and clinicopathologic features. Our findings revealed that negative cytoplasmic expression of ADAMTS14 was significantly associated with lymph node metastasis and AJCC cancer stage. These findings further indicate that ADAMTS14 is involved in the development and progression of OSCC. In addition, the correlations between negative ADAMTS14 protein expression and lymph node metastasis and between ADAMTS14 and AJCC cancer stage indicate that ADAMTS14 could be involved in OSCC metastasis (Table 2).

In human cancers such as breast [56], colon [57], cervical [58], gastric [52], lung cancers [59], leukemia [60], and esophageal squamous cell carcinoma (ESCC), the expression of ADAMTS has been associated with poor prognosis [61]. In the current study, our results show that OSCC patients who presented negative cytoplasmic expression of ADAMTS14 proteins had overall survival times which were notably shorter than those of patients who presented positive cytoplasmic expression of ADAMTS14 (Figure 2). Moreover, univariate analysis revealed that negative ADAMTS14 expression was associated with worse survival, lymph node metastasis, more advanced tumor stages, and higher histological grades in OSCC patients. Multivariate analysis further confirmed that lymph node metastasis, tumor stage, and histological grade were independent prognostic factors of poor OSCC prognosis (Table 3). These results are consistent with those of previous studies and suggest that the ADAMTS family may play a vital role in the development and progression of cancer [52,56,57,58,59,60]. In HCC, ADAMTS14 expression has been associated with more aggressive cancers and poorer clinical outcomes, which are rs12774070 variants in the TSR domain near the glycosylation site, which may affect the TSR domain and alter cell surface association and sequential processing mechanisms [36]. Moreover, interactions between ADAMTS14 gene polymorphism and environmental mutagens is a risk factor for OSCC tumorigenesis and suggests that rs12774070 affects the degree of differentiation of OSCC cells [34].

In summary, results from our study indicate that ADAMTS14 has low expression in OSCC. The downregulation of ADAMTS14 is associated with poor clinicopathological features and may be a useful independent prognostic marker that can help predict the overall survival of OSCC patients. We plan to further validate the results of this study in subsequent experiments that will employ molecular biology and cell biology methods. Nonetheless, the current study provides strong preliminary evidence to suggest that ADAMTS14 can (1) be used as a novel biomarker for OSCC diagnosis and (2) serve as a useful target for OSCC therapy in the future.

## 4. Materials and Methods

### 4.1. Ethics Statement and Tissue Microarrays (TMAs)

The present study analyzed tissues from 250 OSCC patients who were treated at Changhua Christian Hospital, Changhua, Taiwan. All research protocols were approved by the Ethics Committees of Changhua Christian Hospital and adhered to guidelines approved by the Institutional Review Board (IRB150808, date of approval 5 September 2015). TMAs were constructed in the Department of Pathology of Changhua Christian Hospital. Tissue cylinders (2 mm in diameter) were collected from donors and arranged in paraffin that was then made into new FFPE blocks using a homemade, semiautomated tissue array [62]. Sections measuring 4 μm in thickness were cut and attached to glass microscope slides. The core of the perforation included a high number of viable tumor cells with little necrosis from the surrounding or central region. All TMAs were stained with hematoxylin and eosin (H&E), and two senior pathologists confirmed the presence of morphologically representative lesions of the original cancer. Pathological evaluation of tumor stages and histological differentiation were performed according to the American Joint Committee on Cancer (AJCC, 7th Edition) TMN staging system as well as Edmondson and Steiner grading system standards.

### 4.2. Immunohistochemistry Analysis

After deparaffinization and rehydration, TMAs were heated in 0.01 M citrate buffer (pH 6.0) for antigen retrieval, and then incubated in 3% H_2_O_2_ to inhibit endogenous peroxidase activity. ADAMTS14 proteins were detected with polyclonal anti-human ADAMTS14 antibodies (Dilution 1:50X; Catalog number: ab198885; Abcam, Cambridge, MA, USA) at 4 °C overnight, and the LASB 2 kit (Dako, Carpinteria, CA, USA) was used to detect the resulting immune complex. Enzyme activity was visualized using aminoethyl carbazole as a substrate. To determine the specificity of immunohistochemical staining, appropriate positive (known positive cases) and negative (samples that were not incubated with primary antibodies) controls were included at the same time. IHC results were evaluated by two senior pathologists who were blind to assessment scores. Staining intensity for ADAMTS14 protein expression was categorized as being positive or negative, and each category included two subgroups. For the positive expression group, a score of 1+ indicated weak positive protein expression, a score of 2+ indicated moderately positive protein expression and a score of 3+ indicated strongly positive protein expression [63].

### 4.3. Statistical Analysis

Correlations between ADAMTS14 and clinicopathological features were calculated using Fisher’s exact test and the chi-square test. The Kaplan–Meier method was used to determine overall survival curves. Cumulative survival rate was analyzed using the log-rank test. The Cox proportional hazard regression model was employed in univariate and multivariate analyses to confirm prognostic factors [64]. All statistical analyses were carried out using SPSS (version 17) statistical software (SPSS, Inc., Chicago, IL, USA). In all statistical analyses, p values lower than 0.05 were considered statistically significant.

## Figures and Tables

**Table 1:** Demographic and characteristics among oral oral squamous cell carcinoma (OSCC) patients.

Factors	Total Number (*n* = 250)	%
Gender		
Female	12	4.8
Male	238	95.2
Age (Year)		
Range	31–88	
Mean	54.9	
Median	53.0	
T (Tumor size)		
I	60	24.0
II	78	31.2
III	20	8.0
IV	92	36.8
N (Lymph node)		
N0	155	62.0
N1	95	38.0
M (Metastasis)		
No	248	92.2
Yes	2	0.8
AJCC cancer stage		
I	46	18.4
II	54	21.6
III	28	11.2
IV	122	49.0
Histological grade		
Well	42	16.8
Moderate	201	80.4
Poor	7	2.8
Clinical therapy		
Radiotherapy	152	60.8
Chemotherapy	60	24.0

**Table 2:** Clinicopathologic factors associated with ADAMTS14 expression.

Cytoplasmic Staining of ADAMTS14 Total				
Variable	Negative	Positive	(*n* = 250)	*p*-Value
Age	55.23 ± 11.6	55.52 ± 10.6	0.471	
Gender				
Female	3(5.8)	9(4.5)	12	
Male	49(94.2)	189(95.5)	238	0.718 ^a^
Histological grade				
Well	7(13.5)	35(17.7)	42	
Moderate, Poor	45(86.5)	163(82.3)	208	0.469
T status				
T1, T2	24(46.1)	114(57.6)	138	
T3, T4	28(53.8)	84(42.4)	112	0.140
Lymph Node Metastasis				
No	23(44.2)	132(66.7)	155	
Yes	29(55.8)	66(33.3)	95	0.003 **
Distance Metastasis				
No	51(98.1)	197(99.5)	248	
Yes	1(1.9)	1(0.5)	2	0.373 ^a^
Stage				
I, II	14(26.9)	86(43.4)	100	
III, IV	38(73.1)	112(56.6)	150	0.031 *
Drinking				
No	21(45.7)	62(39.0)	83	
Yes	25(54.3)	97(61.0)	122	0.418
Betel nut chewing				
No	16(45.7)	49(39.2)	65	
Yes	19(54.3)	76(60.8)	95	0.488
Survival				
≤3 year	23(44.2)	77(38.9)	100	
>3 year	29(55.8)	121(61.1)	150	0.484
≤5 year	30(57.7)	90(45.5)	120	
>5 year	22(42.3)	108(54.5)	130	0.116

*p*-value by Fisher’s exact test or chi-square test ^a^; * *p* < 0.05; ** *p* < 0.01.

**Table 3:** The effect of clinicopathologic factor and ADAMTS14 expression on mortality density and adjusted hazard ratio among OSCC patients.

Univariate Multivariate.						
Variable	HR	95% CI	*p*-Value	HR	95% CI	*p*-Value
Expression of ADAMTS14						
Negative	1.4			1.2		
Positive	1.0	0.507–0.996	0.047 *	1.0	0.601–1.199	0.353
Lymph Node Metastasis						
No	1.0			1.0		
Yes	1.9	1.451–2.566	<0.001 ***	1.4	1.004–20.42	0.048 *
Stage						
I, II	1.0			1.0		
II, IV	1.8	1.312–2.361	<0.001 ***	1.5	1.065–2.183	0.021 *
Histological grade						
Well	1.0			1.0		
Moderate, Poor	1.9	1.216–2.860	0.004 **	1.9	1.191–2.943	0.007 **

HR (Hazard ratio) was adjusted for gender and age; * *p* < 0.05; ** *p* < 0.01; *** *p* < 0.001.
